# Ultrathin Functional Polymer Modified Graphene for Enhanced Enzymatic Electrochemical Sensing

**DOI:** 10.3390/bios9010016

**Published:** 2019-01-18

**Authors:** Anitha Devadoss, Rhiannan Forsyth, Ryan Bigham, Hina Abbasi, Muhammad Ali, Zari Tehrani, Yufei Liu, Owen. J. Guy

**Affiliations:** 1 Systems and Process Engineering Centre (SPEC), Centre for NanoHealth, College of Engineering, Swansea University, Swansea SA2 8PP, UK; 652686@swansea.ac.uk (R.F.); R.M.Bigham@Swansea.ac.uk (R.B.); H.Y.Abbasi@Swansea.ac.uk (H.A.); 823439@swansea.ac.uk (M.A.); z.tehrani@swansea.ac.uk (Z.T.); 2 Key Laboratory of Optoelectronic Technology & Systems (Chongqing University), Ministry of Education, Chongqing 400044, China; Yufei.Liu@cqu.edu.cn; 3 Centre for Intelligent Sensing Technology, College of Optoelectronic Engineering, Chongqing University, Chongqing 400044, China; 4 Department of Chemistry, College of Science, Swansea University, Swansea SA2 8PP, UK

**Keywords:** graphene, enzyme immobilization, functional polymers, electropolymerization, bio electrochemistry, electrochemical sensing, glucose biosensor, biofunctionalization

## Abstract

Grafting thin polymer layers on graphene enables coupling target biomolecules to graphene surfaces, especially through amide and aldehyde linkages with carboxylic acid and primary amine derivatives, respectively. However, functionalizing monolayer graphene with thin polymer layers without affecting their exceptional electrical properties remains challenging. Herein, we demonstrate the controlled modification of chemical vapor deposition (CVD) grown single layer graphene with ultrathin polymer 1,5-diaminonaphthalene (DAN) layers using the electropolymerization technique. It is observed that the controlled electropolymerization of DAN monomer offers continuous polymer layers with thickness ranging between 5–25 nm. The surface characteristics of pure and polymer modified graphene was examined. As anticipated, the number of surface amine groups increases with increases in the layer thickness. The effects of polymer thickness on the electron transfer rates were studied in detail and a simple route for the estimation of surface coverage of amine groups was demonstrated using the electrochemical analysis. The implications of grafting ultrathin polymer layers on graphene towards horseradish peroxidase (HRP) enzyme immobilization and enzymatic electrochemical sensing of H_2_O_2_ were discussed elaborately.

## 1. Introduction

Rapid detection of low concentration of specific analytes in small sample volumes is critical in the early point-of-care diagnosis. While conventional optical based detection is extremely sensitive [1,2], they are time consuming and require expensive and complex optical imaging devices, sophisticated image recognition software and fluorescence dye labels. Alternatively, electrochemical biosensors utilizing enzyme-modified electrodes—coined as “enzymatic electrochemical biosensors”—have received considerable attention, due to their high sensitivity and specificity. Enzymes are very effective and precise biocatalysts, which perform and regulate certain processes in living systems. The performance of the enzymatic electrochemical biosensors greatly depend on enzyme immobilization, which involves the interaction between the enzyme and the host surface, and thus the surface properties of both plays a significant role in defining their functionality [3,4]. In this context, graphene remains an ideal candidate for hosts due to its large specific surface area and excellent electrical characteristics. Graphene is a single-atom-thick nanomaterial with sp^2^ bonded carbon atoms arranging as a honeycomb structure [5]. The two-dimensional nature of graphene offers numerous remarkable properties such as high carrier mobility, large specific surface area, and excellent electrical conductivity [6,7,8]. For instance, graphene has been exploited for label-free electrochemical sensing due to the high electron transfer rates [9,10,11,12]. Due to the excellent electronic conductivity, graphene based composites have been utilized for improving the biosensor performance [13]. Recently, the graphene field effect transistor (G-FETs) based biosensors are gaining momentum due to its extremely high carrier mobility and capacitance [14,15], where graphene surface is interfaced with various biomolecules and cells. In all these sensors, surface modification is an indispensable step to interface the graphene with biomolecules such as antibodies, cells, enzymes, or single strand DNA probes that can selectively bind/interact to the target biomolecules in solution during biosensor operation. 

A wide range of covalent (with covalent bond formation) and non-covalent (due to van der Waals forces only) functionalization chemistries have been investigated to selectively functionalize the graphene surfaces [16,17,18]. The most common way to functionalize graphene is via vigorous chemical oxidation processes, creating sp^3^ hybridized bonds that would affect the unique electrical properties of graphene [19,20]. Alternative direct covalent functionalization (without oxidation intermediates) methods were developed, however, the low reactivity of graphene basal surface, long reaction time, and low surface coverage limits their practical applications. Conversely, non-covalent routes (pi-stacking) for functionalizing graphene were reported widely in the literature. However, the electrical conductivity of the functionalized graphene has been observed to significantly decrease compared to pure graphene. Moreover, the surface area of the functionalized graphene prepared by covalent and non-covalent techniques decreases significantly due to the destructive chemical oxidation of flake graphite followed by sonication, functionalization, and chemical reduction. Thus, it is critical to develop a non-destructive functionalization route to modify graphene surfaces with engineered sites for binding.

Grafting thin conducting polymer layers have attracted a great deal of attention over the past decade. Electrodes modified with polymer layers can be achieved by different methods, however, electropolymerization is attractive in the preparation of ultra-thin polymer layers because of its experimental simplicity, room temperature utility, degree of dimensional specificity, and precision over the layer thickness via controlling the charge passed and/or the potential at which the deposition is performed. Additionally, the electrochemical potential applied can shift the Fermi level of graphene, increasing its reactivity as compared to direct attack of the covalent sp^2^ bonds with aggressive chemicals. Recently, polydiaminonaphthalene (pDAN), a conductive polymer grafted from aromatic diamines, has been reported to exhibit interesting electrochemical characteristics and compatibility to binding biomolecules and to shorten the distance between the electrode surface and enzyme active sites [21]. Nevertheless, pristine pDAN films exhibit low conductivity at neutral pH, which strongly affects the sensitivity of biosensors. Composites of pDAN with various other materials including carbon nanotubes, gold, Fe, etc., were reported to enhance the performance of the biosensor [21,22,23,24,25]. Recently, Ngyuyen et al. explored the potential application of graphene-pDAN composite for biosensing application [26]. It was reported that the current intensity of Pt electrodes modified with CVD graphene/pDAN is 20 times higher than Pt electrodes modified with pDAN. However, the mechanism of charge transport for pDAN on graphene is a key factor controlling the electrochemical sensing remains unexplored. 

Furthermore, quantification of -NH_2_ groups is critical as it reflects the available active binding sites for the biomolecular immobilization. While a wide range of surface functionalization methods have been reported in literature, to the best of our knowledge, there are only very few articles report on the estimation of amine surface coverage. In 2011, Noel et al. reported the estimation of amino surface densities using calorimetric methods [27]. Later, spectrophotometric based methods involving the intermediate dye formation for the quantification of amine groups onto ceramics [28] and silica surfaces [29]. Shiota et al. has demonstrated the quantification of surface amine moieties on inorganic and/or organic surfaces using cleavable fluorescent compounds [30]. While the method offers good sensitivity, these techniques involve multiple steps. Thus, in this article, we report a controlled deposition of ultrathin functional polymer layers (1,5-diaminonaphthalene) onto CVD graphene surfaces using an electropolymerization technique and evaluated their surface characteristics. The influence of the polymer layer thickness on both the electrochemical properties as well as the enzyme immobilization was evaluated. A simple electrochemical method was demonstrated as a tool for quantifying the surface bound amine groups. Furthermore, the implications of such improved electrochemical performance and/or enzyme immobilization characteristics of ultrathin polymer layer grafted graphene towards enhancing the electrochemical biosensor performance was demonstrated using the classic horseradish peroxidase enzyme, as an example. 

## 2. Materials and Methods

### 2.1. Materials and Reagents

Monolayer chemical vapor deposition (CVD) grown graphene was purchased from Graphenea and the samples were cleaved with 4 × 4 mm dimension. Sulfuric acid (ACS reagent, 95.0–98.0%, Sigma-Aldrich, Dorset, UK), 1,5-diaminonaphthalene (DAN) (99%, Aldrich), ferrocene carboxylic acid (97%, Sigma-Aldrich), phosphate buffered saline tablets (Sigma-Aldrich), horseradish peroxidase enzyme (Sigma), N-Hydroxysuccinimide (NHS) (98%, Sigma-Aldrich), N-(3-Dimethylaminopropyl)-N′-ethylcarbodiimide hydrochloride (EDC) (>99%, Sigma-Aldrich) and H_2_O_2_ (99.5%, Sigma) were used as received. 

### 2.2. Electropolymerization

The polymer films were deposited from 10 mM DAN in 0.25 M H_2_SO_4_ in a three-electrode system, where, monolayer CVD graphene was used as the working electrode, Pt wire as counter electrode and Ag/AgCl served as the reference electrode. The electropolymerization of monomer DAN produces polymer DAN (pDAN) on graphene surface generating NH_2_ groups on the surface. The thicknesses of the pDAN layers were controlled by controlling the number of cycles during the electropolymerization process. 

### 2.3. Surface Functionalization

The surface coverage of amines on pDAN modified CVD graphene was estimated by functionalizing the amine groups with electroactive ferrocene carboxylic acid moieties. Typically, 250 µM ferrocene carboxylic acid was prepared in fresh PBS solution containing 5 mM EDC and NHS, which was incubated at room temperature for 40 min to activate the carboxylic functional group of the redox molecule. The resultant solution was drop-casted on a freshly prepared amine terminated pDAN modified CVD graphene surface and incubated at room temperature for 10 min. The electrode functionalization was performed prior to the electrochemical experiments and was used immediately.

### 2.4. Enzyme Immobilization

Aqueous solution of 5% glutaraldehyde was drop-casted onto the pDAN modified electrodes and was incubated for 30 min. Later, the electrodes were rinsed with de-ionized water (DI-water) and dried under nitrogen. The enzyme immobilization was performed by directly drop-casting 10 µL of horseradish peroxidase (HRP) enzyme (1 mg/mL) onto the modified surface and incubated at 4 °C for 18 h. The electrodes were rinsed with DI-water thrice to remove any weakly bound enzymes. The electrodes were freshly prepared and used immediately following the enzyme immobilization.

### 2.5. Characterization Techniques

The surface topography of pDAN modified graphene electrode was investigated using atomic force microscopy (AFM), (Dimension-3100 Multimode, Bruker, Billerica, MA, USA), and the AFM tip was a silicon-SPM sensor (contact mode), thickness 4 μm, length 125 μm, and width 30 μm. The chemical environment was studied using X-ray photoelectron spectroscopy (XPS). Electrochemical analysis was performed using the advanced potentiostat (PGSTAT-302 from Autolab, Metrohm Autolab, Runcorn, UK) with the scanning voltage in the range of −0.8 to 0.6 V for evaluating the electrochemical performance of enzyme-functionalized electrodes. Standard 3-electrode system was used for the electrochemical evaluation, where CVD graphene was used as the working electrode, Ag/AgCl as the reference electrode and Pt wire as the counter electrode. 0.01 M phosphate buffer saline (PBS) solution (pH = 7.4) was used as the electrode unless otherwise stated. 

## 3. Results and Discussion

### 3.1. Electropolymerization

**Figure 1:**
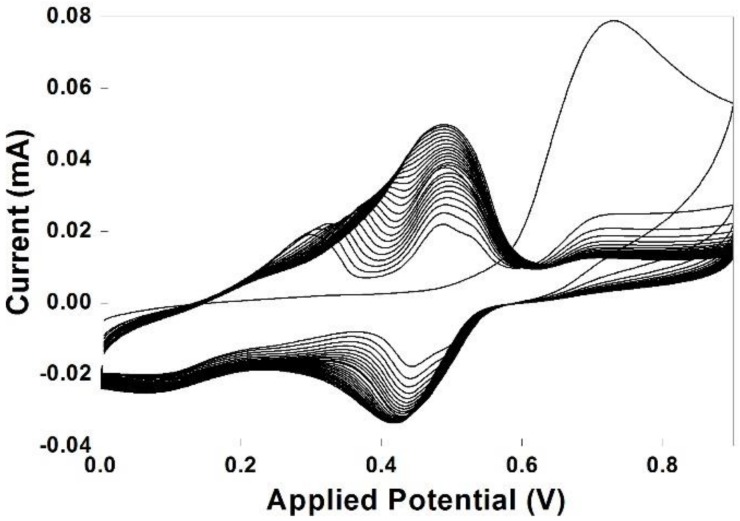
Electropolymerization of 10 mM 1,5-daminonaphthalene in 0.25 M H_2_SO_4_ at a scan rate of 50 mVs^−1^ over 25 cycles. The electropolymerization was carried at room temperature.

Cyclic voltammogram for the electropolymerization of 10 mM 1,5-DAN on monolayer CVD graphene at a sweep rate of 50 mVs^−1^ is shown in Figure 1. A well-defined anodic irreversible peak at 0.71 V (vs. Ag/AgCl) originated during the first cycle confirms the monomer aromatic diamine oxidation. The oxidation peak at 0.71 V mainly corresponds to the oxidation of the monomers. which was followed by the growth of the polymer with the monomer oxidation peak suppressed in subsequent potential cycles. This behavior attests to the autocatalytic growth of conducting polymers [31], in which the oxidized polymer acts as an electrocatalyst for taking electrons away from the monomer molecules, leading to the polymer growth. The oxidation at 0.71 V is partially associated with the oxidation of amine groups from the monomer, which would allow the polymer growth via binding with one amine group per monomer, thus leaving the other amine group free for subsequent biomolecule binding [32]. No cathodic peak corresponding to the irreversible anodic peak at 0.71 V indicates a faster consumption of the electrogenerated cation radicals by follow-up polymerization reactions on the electrode. From the second cycle, two monomer oxidation peaks were observed at about 0.31 and 0.47 V, indicating the initial stages of electropolymerization. The increase in current density evidences the formation of polymeric film on monolayer CVD graphene surface. Meanwhile, the monomer oxidation peak at 0.71 V gets suppressed gradually and finally disappears indicating the predominant polymer growth process consuming the monomers. Furthermore, with an increase in scan cycles, the two monomer oxidation peaks at 0.31 V and 0.47 V were merged at 0.5 V.

### 3.2. Surface Analysis

**Figure 2:**
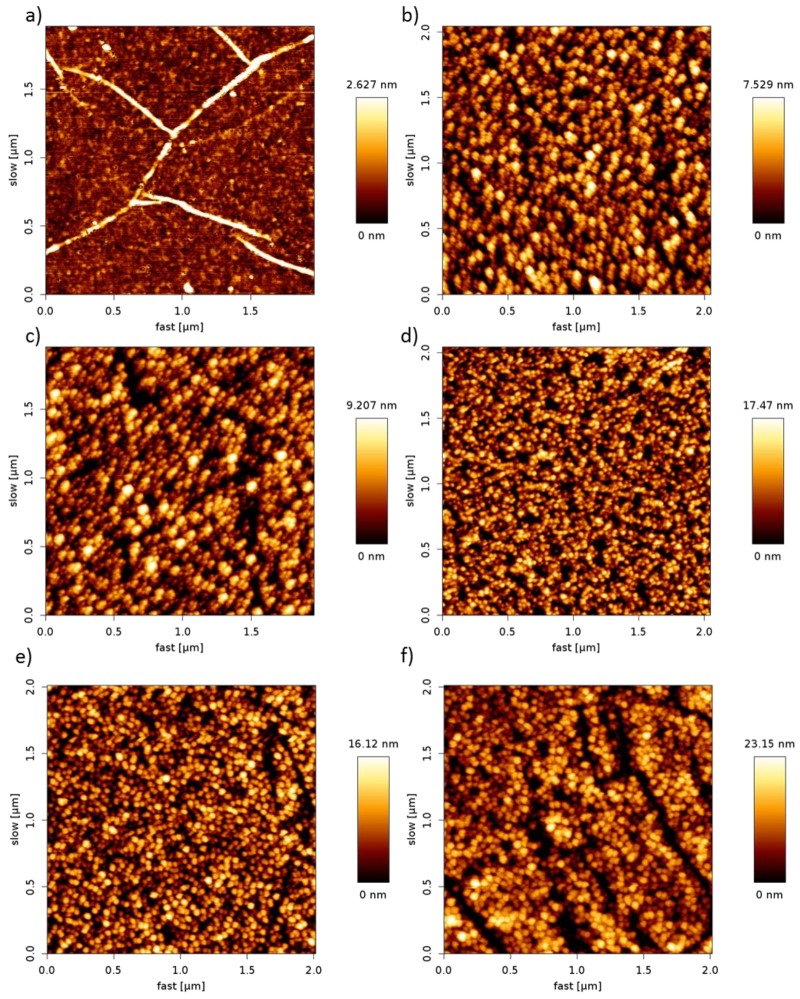
AFM images of pDAN layers deposited on CVD graphene surface at different number of cycles: (**a**) 0, (**b**) 5, (**c**) 10, (**d**) 25, (**e**) 50, and (**f**) 75 cycles, respectively, at a scan rate of 50 mVs^−1^.

The surface topography of the pDAN functionalized graphene was analyzed using atomic force microscopy (AFM) analysis. Figure 2 shows the AFM images of un-modified graphene (a) and pDAN modified CVD graphene (b–f) with different scan cycles. It is found that the electropolymerization process produces a good coverage ultra-thin polymer layers on CVD graphene. The surface roughness of the pDAN films increases with increases in the scan cycles, confirming the formation of thicker layers at higher scan cycles. The surface thickness of the pDAN layers were measured using scanning electron microscopy (Supporting Information; Figure S1), which confirms the increase in thickness from 25 ± 2 nm to 53 ± 5 nm upon increasing the number of scan cycles from 25 to 75 cycles. 

### 3.3. Chemical Environment Analysis

**Figure 3:**
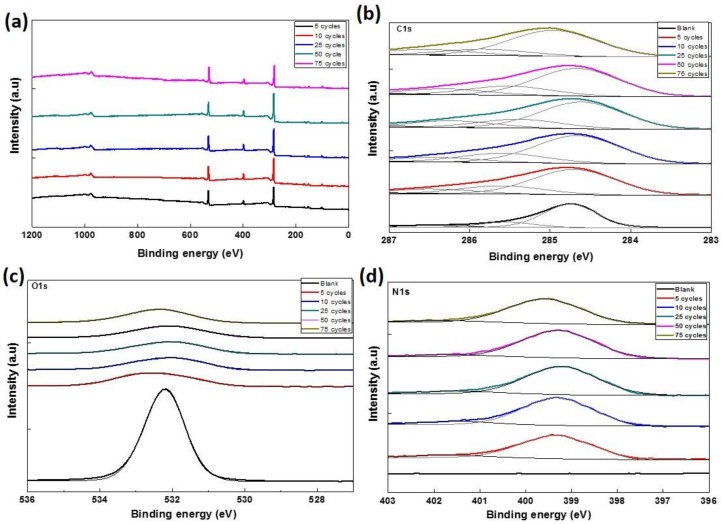
XPS spectrum showing (**a**) wide scan, (**b**) C 1s, (**c**) O 1s, and (**d**) N 1s spectrum of CVD graphene and pDAN functionalized graphene at different scan cycles.

X-ray photoelectron spectroscopic measurements were carried out on CVD graphene and pDAN modified CVD graphene surfaces (Figure 3) to study the chemical environment at the modified surfaces. As presented in Figure 3a, the XPS was analyzed from 0 to 1200 eV, which is the full range of binding energy area to find out the main atomic components of the graphene. C, O, and Si were detected in all the samples. The wide scan acquisition for pristine graphene shows typical C1s peak at 283.7 eV. The Si peaks at 153.7 eV (Si 2s) and 103.63 eV (Si 2p) are originally from the SiO_2_/Si substrate; whereas, the O peaks might be originating from both SiO_2_/Si substrate as well as graphene (edges and defects of the graphene lattice had bonds with oxygen). The atomic percentage of C, O, and N were determined from the XPS spectra and are presented in Table 1. It is observed that the atomic percentage of C and N increases drastically upon electropolymerization, which can be ascribed to the carbon and nitrogen species of DAN. Whereas the atomic percentage of O decreased significantly upon pDAN layer formation, indicating that the predominant O species of pristine CVD graphene were originated from the SiO_2_/Si substrate, which might be masked by the pDAN layer (>5 nm). To further analyze the nature of C, O, and N species, the C 1s (Figure 3b), O 1s (Figure 3c), and N 1s (Figure 3d) peaks were decomposed into Gaussian/Lorentzian multi-peaks to analyze the distribution of C–C/C–O/C=O bonding. The C 1s peak of pDAN modified graphene was deconvoluted into four carbon peaks, which are ascribed to C_aromatic_ (~284.8 eV), C_aliphatic_ (~285.4 eV), C=C-N/C-NH_2_/C-O (~286.5 eV), and C=O/C=N (~287.5 eV) [33]. The N 1s peak of pDAN modified graphene was observed at 399 eV, indicating that nitrogen moieties were introduced upon electropolymerization. Furthermore, the two deconvoluted peaks at ~399.2 eV and ~401.5 eV corresponds to C-NH- and C-NH_2_^+^, respectively [34], confirming that the pDAN electropolymerization process generates -NH_2_ groups on the graphene surface. 

### 3.4. Electrochemical Analysis

**Figure 4:**
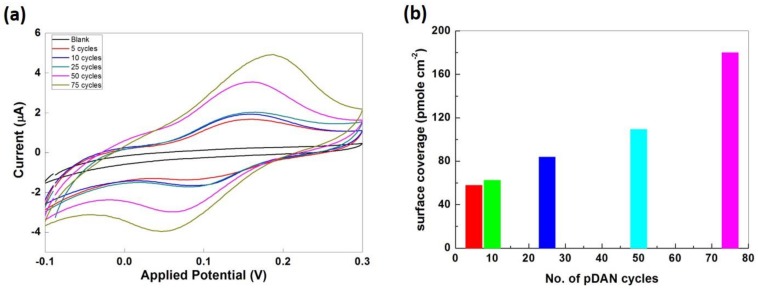
(**a**) Cyclic voltammogram of Ferrocene carboxylic acid functionalized CVD graphene and pDAN modified CVD graphene at different scan cycles. PBS was used as the electrolyte and the CVs were recorded at a 5 mVs^−1^ scan rate. (**b**) Bar chart showing the amine surface coverage in picomole cm^−2^ vs. number of pDAN scan cycles.

The surface coverage of amine groups on pDAN modified CVD graphene was estimated via functionalizing the electrodes with an electroactive ferro-centered molecule, ferrocene carboxylic acid, using a standard EDC/NHS coupling reaction. Ferrocene carboxylic acid functionalized electrodes were scanned between −0.1 V and 0.3 V at 5 mVs^−1^ sweep rate. Figure 4 shows the cyclic voltammogram of ferrocene carboxylic acid functionalized CVD graphene and pDAN modified CVD graphene electrodes. As expected, no redox peak was found in CVD graphene upon ferrocene carboxylic acid functionalization due to the lack of an amine functional group on the surface, confirming the lack of physisorption and thus attesting the robustness of this method. Strong redox peaks were observed at 0.15 V and 0.05 V at ferrocene carboxylic acid functionalized pDAN modified CVD graphene electrodes, which corresponds to the oxidation and reduction of ferrocene moieties. The surface coverage of the ferrocene was estimated using the standard formula:

Г = Q/nFA(1)

where Q is the charge obtained by integrating the anodic peak at a low scan rate (coulomb), n is the number of electrons transferred, F is the Faraday constant (96485 sA Mol^−1^), and A is the geometrical area of the electrode (cm^2^). The surface coverage of ferrocene moieties were found to be 58.04, 62.60, 83.95, 109.52, and 179.99 pmol cm^−2^ for the electrodes modified with 5, 10, 25, 50, and 75 cycles of pDAN layers, respectively. Assuming an effective binding (100%), these surface coverage’s reflect the amine surface coverage on pDAN modified CVD graphene substrates. Thus, the amine surface coverage increases with an increase in the thickness of the pDAN layer. 

**Figure 5:**
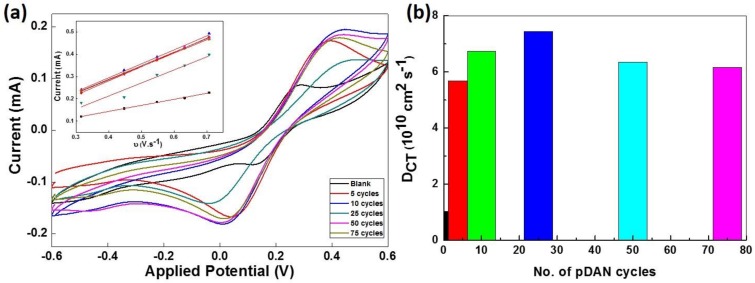
(**a**) Cyclic voltammogram of CVD graphene and pDAN modified CVD graphene with different scan cycles in 5 mM K_3_[Fe(CN)_6_] in PBS at a scan rate of 50 mVs^−1^. Inset shows the linear dependency of the peak current vs. square root of scan rate for CVD graphene and pDAN modified CVD graphene at different scan cycles. (**b**) Bar chart illustrating the change in charge transfer diffusion coefficient vs. number of pDAN scan cycles.

The electro activity of as-deposited pDAN films were studied using the cyclic voltammetry. Figure 5 shows the voltammetry response of pDAN films in 5 mM Fe^2+^/Fe^3+^ redox couple at scan rates 2–200 mVs^−1^. The pristine graphene modified electrode shows the typical oxidation and reduction peaks at 0.25 V and 0.1 V, which is ascribed to Fe^2+^/Fe^3+^ redox reaction. However, the pDAN modified graphene electrodes showed that the redox potentials moved to a more positive value (0.5 V and 0.042 V), indicating the occurrence of additional chemical reactions. The peak current i_pc_ is linear with the square root of scan rate (inset of Figure 5), indicating a typical surface confined redox behavior. 

The charge transfer diffusion coefficient, D_CT_, was calculated using Randles-Sevcik equation [35].

i_p_ = (2.69 × 10^5^) n^3/2^ A C D^1/2^ ʋ^1/2^ (2)

where i_p_ represents the peak current, n is the number of electrons transferred (n = 1), A is the area of the electrode, D is the charge transfer diffusion coefficient, C is the concentration of redox couple (5 mM), and υ is the scan rate. The pristine CVD graphene electrodes shows a typical D_CT_ of 1.023 × 10^10^ cm^2^ s^−1^ (Figure 5b). It is found that the charge transfer diffusion coefficient increases with an increase in the pDAN layer thickness and reaches the maximum (7.445 × 10^10^ cm^2^ s^−1^) around 25 cycles, after which the D_CT_ decreases. This confirms that the electron transfer rates are affected by the thickness of the pDAN layer (as shown in Figure S2), and thus, it is significant to identify the optimal thickness. While an increase in the amine surface coverage might improve the enzyme immobilization via offering additional binding sites, there exists a trade-off between the amine surface coverage and the maximum charge transfer characteristics. Thus, it is critical to explore the sweet spot for higher surface coverage without compromising the charge transfer characteristics to achieve improved sensor performance. 

### 3.5. Enzymatic Electrochemical Sensing

**Figure 6:**
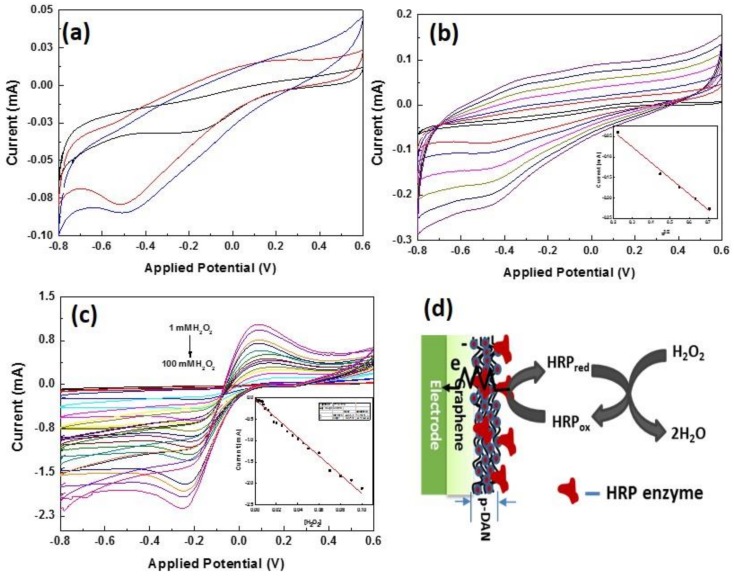
(**a**) Cyclic voltammogram of HRP functionalized CVD graphene (black), pDAN modified CVD graphene with 5 cycles (red) and 25 cycles (blue) in PBS at a scan rate of 50 mVs^−1^. (**b**) Cyclic voltammograms of HRP functionalized CVD graphene modified with 25 cycles of pDAN at different scan rates (50–500 mVs^−1^) in PBS. Inset shows the linear dependency of the peak current vs. square root of scan rate. (**c**) Electrochemical H_2_O_2_ sensing at HRP functionalized CVD graphene/25pDAN modified electrode. Inset shows the current response at −0.25 V vs. concentration of H_2_O_2_. (**d**) Schematic representation of the electrochemical sensing mechanism at HRP-enzyme functionalized CVD graphene/25pDAN modified electrode.

Based on the initial electrochemical characterization, the electrodes modified with 25 cycles of the pDAN layer were chosen to be the optimized electrode for sensing. The electrodes were functionalized with HRP enzyme. HRP is an electrochemically active enzyme, which has heme as an active site, and the heme-iron oxidation state is HRP-Fe(III) in the ground state [36]. The native HRP-Fe(III) can be directly reduced at the electrode surface to HRP-Fe(II) by one e-transformation [37]. Figure 6a shows the electrochemical response of HRP modified CVD graphene (black), pDAN modified CVD graphene with 5 cycles (red), and 25 cycles (blue) in PBS at a scan rate of 50 mVs^−1^. 

The surface coverage of immobilized HRP enzyme on the electrodes can be estimated using the reduction peaks. As anticipated, the redox peaks of heme protein increases with an increase in the pDAN cycles indicating higher loading of HRP enzymes upon pDAN modification. From the integration of the reduction peaks of HRP functionalized modified graphene electrodes at 50 mVs^−1^, the surface coverage of HRP enzyme was found to be 9.5617 × 10^−10^ mol·cm^−2^, 2.43 × 10^−9^ mol·cm^−2^, and 3.0026 × 10^−9^ mol·cm^−2^ for HRP immobilized on pristine CVD graphene, 5pDAN layers and 25pDAN layers modified CVD graphene electrodes, respectively. It is found that the pDAN surface modifications increases the enzyme immobilization that could be ascribed to the increased binding sites upon polymer grafting. Additionally, the enzyme surface obtained via pDAN modifications shows higher enzyme loading that the previously reported values [38,39,40]. Figure 6b shows the electrochemical response of HRP functionalized CVD graphene modified with 25 cycles of pDAN at different scan rates (50–500 mVs^−1^) in PBS. The linear change in the cathodic peak current with square root of scan rate given in the inset of Figure 6b, suggests a surface confined, quasi-reversible electron transfer process (R^2^ = 0.98985). The enzymatic electrochemical sensor performance of the modified electrodes (Figure 6c) were studied at different concentrations of H_2_O_2_. It is clear from Figure 6c that the redox peak intensities at 0.2 V and −0.25 V increases with increases in the concentration of H_2_O_2_. The inset figure shows the current response of the electrodes (@−0.25 V) at different concentrations of H_2_O_2_. This increase in the redox current can be ascribed to the increased electron transfer between the heme protein HRP and the electrode upon the reduction of H_2_O_2_ into H_2_O, as shown in Figure 6d. It is found that 25 cycles pDAN modified CVD graphene electrodes shows a wide linear range between 1 × 10^−3^ M to 100 × 10^−3^ M with 100 × 10^−6^ M limit of detection compared to earlier reports [41,42,43,44]. The wide linear detection range from our work could be ascribed to the synergistic effects of increased electron transfer characteristics, as well as higher enzyme loading, upon introducing thin polymer layers. 

## 4. Conclusions

A controlled deposition of 1,5-diaminonaphthalene (DAN) was achieved on CVD graphene surfaces using direct electropolymerization process. The surface morphology analysis of pristine and pDAN modified CVD graphene shows uniform deposition of the polymer layer on CVD graphene. The XPS analysis shows that the atomic percentage of C increases from 25.64% to 71.76%, whilst the atomic percentage of N drastically improves from 0 to 9.12%. Additionally, it is observed from the XPS analysis that the increase in the N atomic percentage corresponds to the formation of new -NH_2_ peaks upon electropolymerization. The electrochemical analysis showcase that the pDAN polymers exhibit thickness dependent electron transfer phenomenon. A simple electrochemical tool was demonstrated for the estimation of total amine surface coverage, which can be adapted for other similar systems. The enzymatic electrochemical sensing ability of these electrodes were demonstrated using a standard H_2_O_2_ detection system via functionalizing the HRP. The experimental results evidence that the electrochemical sensing efficiency increases, owing to the synergistic effects of better electron transfer as well as higher enzyme loading on pDAN modified electrodes.

## Figures and Tables

**Table 1:** Carbon, oxygen and nitrogen atomic percentages for untreated and pDAN functionalized CVD graphene. Atomic percentages were determined using XPS analysis.

Samples.	Atomic Percentage (%)		
	C	O	N
Graphene	25.64	49.47	0.00
5 cycles pDAN	66.86	17.67	8.61
10 cycles pDAN	71.14	14.65	9.64
25 cycles pDAN	71.77	14.39	9.89
50 cycles pDAN	72.90	14.04	9.55
75 cycles pDAN	71.76	14.90	9.12

## Supplementary Materials

The following are available online at http://www.mdpi.com/2079-6374/9/1/16/s1, Figure S1: SEM images representing (a) top view of pDAN modified CVD graphene and the cross-sectional images of CVD graphene electrodes modified with (b) 25 cycles; (c) 50 cycles and (d) 75 cycles, Figure S2: Electrochemical Impedance spectrum of CVD graphene and CVD graphene modified with 25 cycles of pDAN polymer.
