# Supplementary material for: Ultrathin Functional Polymer Modified Graphene for Enhanced Enzymatic Electrochemical Sensing
Source: Biosensors (Basel). 2019 Jan 18;9(1):16. doi: 10.3390/bios9010016 (PMC6468408; doi:10.3390/bios9010016)
Supplement: Supplementary file 1 [file biosensors-09-00016-s001.pdf]

Supporting Information

Ultrathin functional polymer modified graphene for enhanced enzymatic electrochemical sensing

Anitha Devadoss,[1*] Rhiannan Forsyth,[1] Ryan Bigham,[1] Hina Abbasi,[1] Muhammad Ali,[1] Zari Tehrani,[1] Yufei Liu,[2,3] and Owen. J. Guy[1,4*]

[1] Systems and Process Engineering Centre (SPEC), Centre for NanoHealth, College of Engineering, Swansea University, Swansea, SA2 8PP, UK.

[2] Key Laboratory of Optoelectronic Technology & Systems (Chongqing University), Ministry of Education , Chongqing 400044, China

[3] Centre for Intelligent Sensing Technology, College of Optoelectronic Engineering, Chongqing University, Chongqing 400044, China

[4] Department of Chemistry, College of Science, Swansea University, Swansea, SA2 8PP, UK

* Correspondence: anitha.devadoss@swansea.ac.uk; Tel.: +44-179-260-6475

SEM Analysis:

The surface thickness of both untreated and pDAN modified CVD graphene working electrodes were analyzed using scanning electron microscopy (SEM) measurements. Fig. S1 shows the SEM images of pDAN modified CVD graphene.

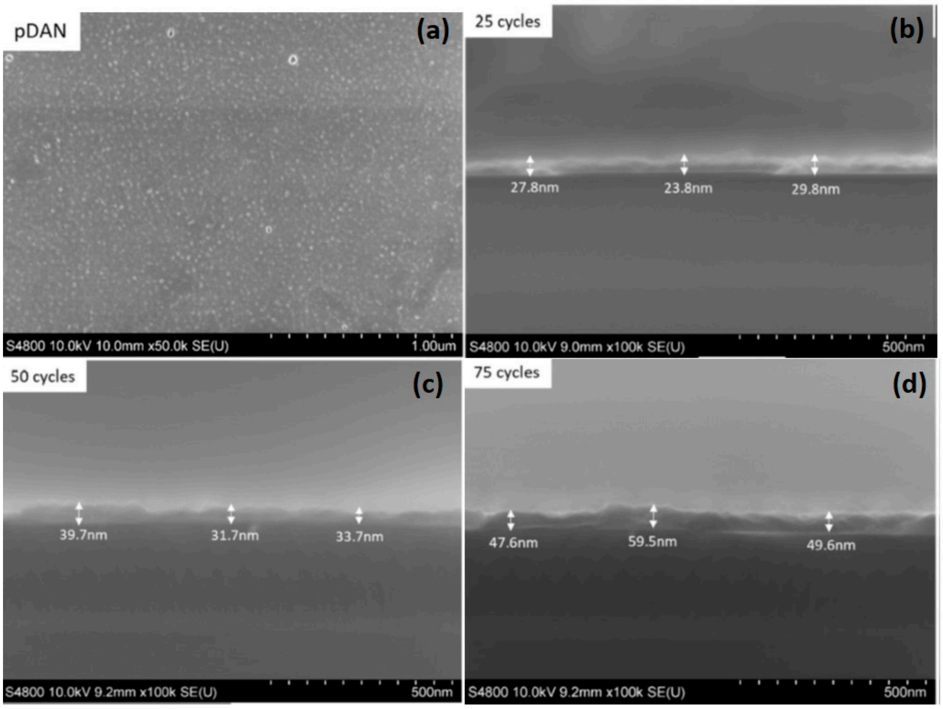

Fig. S1: SEM images representing (a) top view of pDAN modified CVD graphene and the cross-sectional images of CVD graphene electrodes modified with (b) 25 cycles; (c) 50 cycles and (d) 75 cycles.

Fig. S1 shows the top view of pDAN modified CVD graphene sample and Fig. S1 (a-c) shows the cross-sectional images of pDAN modified CVD graphene samples at 25, 50 and 75 cycles respectively. It is clear that the SEM images confirm an increase in thickness from 25±2 nm to 53±5 nm upon increasing the number of scan cycles from 25 to 75 cycles.

Electrochemical Impedance Analysis:

Fig. S2 shows the electrochemical impedance spectrum of highly oriented pyrolytic graphene electrode (HOPG) and pDAN modified HOPG. It is clear from the Fig. S2, that the charge transfer resistance of HOPG electrode gets reduced upon polymerization. The charge transfer resistance of pristine HOPG was found to be around 3000 ohms, which gets reduced to 600 ohms upon grafting ultra-thin polymer layers, which could be attributed to the increased charge transfer rates – as observed from electrochemical analysis.

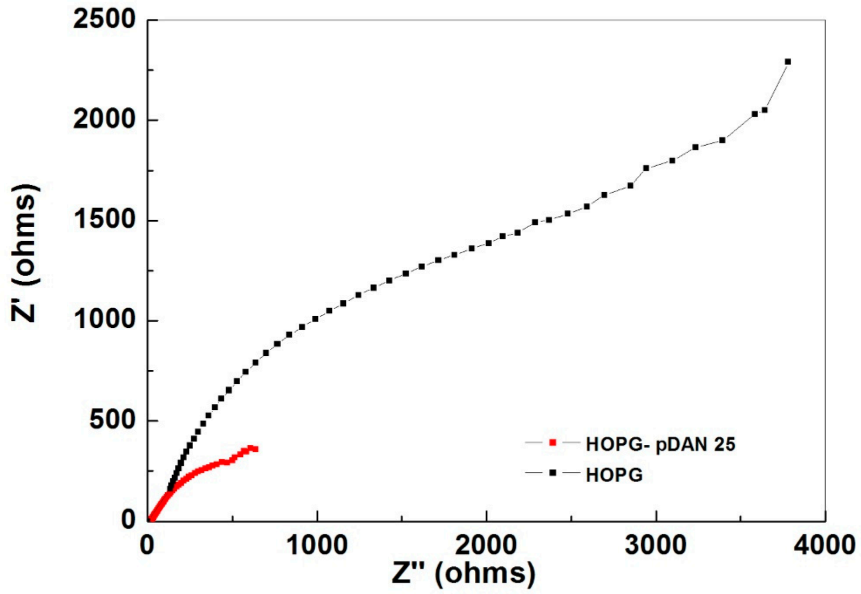

Fig. S2: Electrochemical Impedance spectrum of CVD graphene and CVD graphene modified with 25 cycles of pDAN polymer.
